# Is there a role for IGF‐1 in the development of second primary cancers?

**DOI:** 10.1002/cam4.871

**Published:** 2016-10-13

**Authors:** Thurkaa Shanmugalingam, Cecilia Bosco, Anne J. Ridley, Mieke Van Hemelrijck

**Affiliations:** ^1^ Division of Cancer StudiesCancer Epidemiology GroupKing's College LondonLondonUnited Kingdom; ^2^ Randall Division of Cell and Molecular BiophysicsKing's College LondonLondonUnited Kingdom

**Keywords:** Breast cancer, colorectal cancer, IGF‐1, lung cancer, prostate cancer, second primary cancer

## Abstract

Cancer survival rates are increasing, and as a result, more cancer survivors are exposed to the risk of developing a second primary cancer (SPC). It has been hypothesized that one of the underlying mechanisms for this risk could be mediated by variations in insulin‐like growth factor‐1 (IGF‐1). This review summarizes the current epidemiological evidence to identify whether IGF‐1 plays a role in the development of SPCs. IGF‐1 is known to promote cancer development by inhibiting apoptosis and stimulating cell proliferation. Epidemiological studies have reported a positive association between circulating IGF‐1 levels and various primary cancers, such as breast, colorectal, and prostate cancer. The role of IGF‐1 in increasing SPC risk has been explored less. Nonetheless, several experimental studies have observed a deregulation of the IGF‐1 pathway, which may explain the association between IGF‐1 and SPCs. Thus, measuring serum IGF‐1 may serve as a useful marker in assessing the risk of SPCs, and therefore, more translational experimental and epidemiological studies are needed to further disentangle the role of IGF‐1 in the development of specific SPCs.

## Introduction

Within the last 40 years, cancer survival rates have doubled in the UK 1, mainly due to advances in treatment, and the increased detection of cancer at an early stage 2. In England and Wales, approximately 50% of adult cancer patients diagnosed in 2010 to 2011 are predicted to survive 10 years or more 1. In the US, the 5‐year relative survival rate for all cancers diagnosed in 2004–2010 was 68%, an increase from 49% in 1975–1977 3. Side‐effects of cancer treatment and possible underlying etiological mechanisms, such as IGF‐1 metabolism, are thought to be implicated in the development of second primary cancers (SPCs). Therefore, identifying which cancer survivors have a high risk of developing SPCs is crucial.

It is well known that smoking 4, 5, obesity 6, 7, and insulin resistance 8, 9 are risk factors for the development of first primary cancers. However, the role of these risk factors in the development of SPCs in cancer survivors is less clear. There is some evidence that SPCs may be the result of genetic and hormonal risk factors 10, 11, 12, of late effects of chemo‐ and radiotherapy 2, 13, smoking and alcohol effects 14, 15, as well as nonmodifiable variables such as age and gender. For instance, a study based on the Swedish Family‐Cancer Database concluded that, compared with the general population, males and females diagnosed with an initial primary cancer were 1.3–1.6 times more likely to develop a second cancer, respectively 16.

Epidemiological evidence suggests that survivors of certain types of cancers have a higher risk of developing SPCs. For example, contralateral breast cancer is the most common SPC that develops in patients diagnosed with a first breast cancer, accounting for approximately 50% of all SPCs 17. Furthermore, breast cancer has emerged as the most common solid cancer among female survivors of Hodgkin's lymphoma (diagnosed in childhood), which is largely due to the high‐dose chest irradiation for Hodgkin's lymphoma 2, 18. In addition, urological cancers (bladder, kidney, testes, and penile cancers) are consistently more prevalent among men with prostate cancer 19. Indeed, it has been hypothesized that variations in the insulin‐like growth factor (IGF) pathway, specifically IGF‐1 and its binding protein 3 (IGFBP‐3), could account for the increased risk of SPCs 20.

Recently, several studies have identified IGF‐1 to be associated with an increased risk of developing a number of common cancers, including lung 21, breast 22, colorectal 23, and prostate 24. Circulating levels of IGF‐1 have been linked to the development of SPCs in men with head and neck squamous cell carcinoma 20. However, so far a role of IGF‐1 in development of SPCs following diagnosis of prostate cancer, breast cancer, colorectal cancer, or lung cancer has not been analyzed. In addition, a disorder known as Laron syndrome which is associated with low circulating levels of IGF‐1 and IGFBP‐3 25 are protected from developing cancer, but instead can develop diabetes and cardiovascular disease 26.

With the increase in number of cancer survivors, the long‐term health outcomes of this population need to be carefully examined. Approximately, one in five cancers is diagnosed in those with a previous diagnosis of cancer, and hence, these “second primary cancers” are a leading cause of morbidity and mortality among cancer survivors 27. It is therefore of interest to investigate the role of IGF‐1 in the development of various SPCs as it can help us understand the potential underlying mechanism for carcinogenesis. This review therefore aims to identify whether IGF‐1 plays a role in the development of SPCs, by assessing epidemiological evidence available to date.

## Literature Review

We used a computerized literature search database (PubMed and EMBASE) to identify full text and abstract studies of English language, using human subjects and published between the years 1999 and 2015. Searches were performed with and without the Medical Subject Heading (MeSH) terms for “cancer”, “breast cancer”, “lung cancer”, “prostate cancer”, “colorectal cancer”, and “meta analysis”, combined with the keywords “second primary cancer” and “IGF 1”. All references of the selected articles were checked using hand searches.

## IGF‐1 in First Primary Cancers

This section provides an overview of evidence for the emerging role of IGF‐1 in the development of first primary cancers, with a focus on epidemiological studies (Table 1) as well as experimental studies investigating the underlying biological mechanisms.

**Table 1:** Studies of cancer risk related to IGF‐1 level

Author (Year)	Control (*n*)	Cases (*n*)	Cancer risk related to IGF‐1 level	Reference
Breast cancer				
Peyrat (1993)	92	44	Median concentrations: 26 ng/mL (BCa) versus 20 ng/mL (controls)	38
Endogenous Hormones and Breast Cancer Collaborative Group (2010)	1839	1032	OR for BCa in the highest versus lowest fifth of IGF1 concentration was 1.28 (95% CI: 1.14–1.44; *P* < 0.0001)	39
Rinaldi (2006)	312	202	Highest versus lowest quintile OR 1.38 (95% CI: 1.02–1.86; *P* = 0.01) for women who develop breast cancer after 50 years of age	40
Kaaks (2014)	259	193	OR=1.41 (95% CI: 1.01–1.98; *P* = 0.01 for the highest versus lowest quartile, for ER+ breast tumors overall (pre‐ and postmenopausal women combined)	41
Baglietto (2007)	4296^a^	119	HR for BCa comparing the fourth with the first quartiles was 1.20 (95% CI: 0.87–1.65).	42
	1954^b^ versus 736	68 versus 9	HR for BCa in older women comparing the fourth with the first quartiles (+60 years) was 1.61 (95% CI: 1.04–2.51) versus 0.60 (95% CI: 0.25–1.45) for younger women (<50 years)	
Renehan (2004)	Meta‐analysis of 4 studies	Meta‐analysis of 4 studies	High concentrations of IGF‐1 were associated with an increased risk of premenopausal BCa (OR comparing 75th with 25th percentile 1.65, 95% CI: 1.26–2.08; *P* < 0.001)	44
Shi (2004)	1306	779	Premenopausal women: Nearly 40% increase in BCa risk among those who had higher IGF‐1 in the circulation (overall OR 1.39, 95% CI: 1.16–1.66).	45
	1552	911	No association in postmenopausal women (overall OR 0.93, 95% CI: 0.80–1.10).	
Sugumar (2004)	1471	764	Subjects with higher circulating levels of IGF‐1 had increased risk of premenopausal BCa with an OR of 1.74 (95% CI: 0.97–3.13; *P* = 0.06)	46
Schernhammer (2006)	158	79	RR for top versus bottom quartile of IGF‐1 was 0.98 (95% CI: 0.69–1.39; *P* = 0.77)	47
Hankinson (1998)	92	46	Postmenopausal women: No association between IGF‐1 concentrations and BCa risk (top vs. bottom quintile of IGF‐1, RR = 0.85 [95% CI: 0.53–1.39]).	22
	35	35	RR of BCa among premenopausal women by IGF‐1 concentration (top vs. bottom tertile) was 2.33 (95% CI: 1.06–5.16; *P* = 0.08)	
Lung cancer				
Ahn (2006)	101	38	OR for LCa risk by IGF‐1 concentrations (highest vs. lowest quartile) was 0.69 (95% CI: 0.41–1.15); *P* = 0.26	51
London (2002)	159	51	OR for LCa risk by IGF‐1 concentrations (highest vs. lowest quartile) was 0.73 (95% CI: 0.43–1.24); *P* = 0.80	53
Lukanova (2001)	47	23	OR for LCa risk by IGF‐1 concentrations (highest vs. lowest quartile) was 0.79 (95% CI: 0.29–2.19); *P* = 0.53	54
Morris (2006)	11,072	843	Meta‐analysis: OR for LCa risk by IGF‐1 concentrations (highest vs. lowest quartile) was 1.02 (95% CI: 0.80–1.31); *P* = 0.64	55
Yu (1999)	54	74	High plasma levels of IGF‐1 were associated with an increased risk of LCa (OR = 2.06; 95% CI: 1.19–3.56; *P* = 0.01)	21
Chen (2009)	Meta‐analysis of 6 studies	Meta‐analysis of 6 studies	Pooled OR for LCa risk by IGF‐1 concentrations (highest vs. lowest quartile) was 0.87 (95% CI: 0.60–1.13)	56
Cao (2012)	Meta‐analysis of 6 studies	Meta‐analysis of 6 studies	OR for LCa risk by IGF‐1 concentrations (highest vs. lowest quartile) was 1.05 (95% CI: 0.80–1.37); *P* = 0.74	57
Prostate cancer				
Mantzoros (1997)	52	51	Increment of 60 ng mL corresponded to an OR of 1.91 (95% CI: 1.00–3.73; *P* = 0.05)	62
Colorectal cancer				
Nomura (2003)	282	177 (colon cancer)105 (rectal cancer)	Weakly positive association of IGF‐I with colon cancer. Colon cancer cases in third (IGF‐1 of 137–174 ng/mL) and fourth quartile (IGF‐1 > 174 ng/mL) had increased risk compared with controls (OR of 2.2 and 1.8, respectively)No association of IGFI with rectal cancer	68
Palmqvist (2002)	336	110 (colon cancer)58 (rectal cancer	Increase in colon cancer risk with increasing levels of IGF‐1 (OR of 2.30 and 2.66 for third and fourth quartile, respectively)Rectal cancer risk was inversely related to levels of IGF‐1 (OR of 0.33 and 0.33 for third and fourth quartile, respectively)	69
Tripkovic (2007)	52	52	Increase in IGF‐1 level was followed by a 3.15‐fold increased risk for developing colon cancer with levels of IGF‐1 > 310 ng/mL, whereas twice as many controls exhibited levels of IGF‐1 < 107 ng/mL	70
Ma (1999)	318	193	Men in the highest quintile for IGF‐I had an increased risk of colorectal cancer compared with men in the lowest quintile (RR = 2.51; 95% CI: 1.15–5.46; *P* = 0.02)	71
Kaaks (2000)	200	102	Colorectal cancer risk showed a modest but statistically nonsignificant positive association with levels of IGF‐I	72

BCa, breast cancer; OR, odds ratio; CI, confidence intervals; HR, hazard ratios; RR, relative risk; LCa, lung cancer.

a Breast cancer cases and person‐years calculated from the 2284 women with IGF‐I measured.

b Breast cancer cases and person‐years.

**Figure 1:**
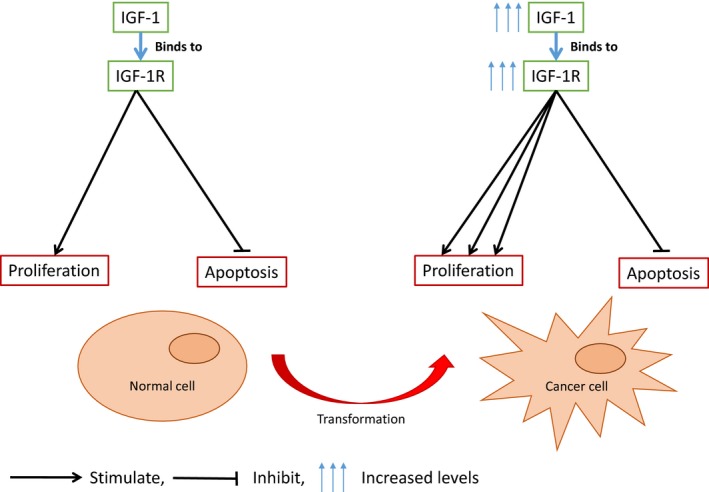
The effects of IGF‐1 and IGF‐1R on normal and cancerous cells.

IGF‐1 is a single‐chain polypeptide growth factor 28, 29, 30 that is related to insulin and IGF‐2 31. IGF‐1 stimulates cell growth, proliferation, and differentiation, and is essential for normal organismal growth and development 32, 33. IGF‐1 binds to the insulin‐like growth factor 1 receptor (IGF‐1R), which is a tyrosine kinase receptor 34. IGF‐1 has a higher binding affinity than IGF‐2 for IGF‐1R. IGF‐1R initiates a cascade of downstream signal transduction pathways known to be involved in cell growth, proliferation, and cancer, including Ras/Raf/ERK and PI3K/Akt/mTOR 35. The majority of IGF‐1 found in the circulation is produced by the liver, functioning as an endocrine hormone. IGF‐1 is also produced in other organs where autocrine or paracrine mechanisms have a role 36. Ample evidence indicates that IGF‐1 and IGF‐1R are important for growth and survival of cancer cells 37, 38 (Fig. 1). The expression of the IGF‐1 gene is primarily regulated by growth hormone (GH), and to a smaller extent by various other hormones 35. By contrast, IGF‐1 that is synthesized locally in an autocrine or paracrine manner may stimulate growth of some cancers 36. The circulating levels of IGF‐1 change markedly with age, peaking at puberty, and slowly declining with increasing age; this fluctuation is regulated by GH, which itself has mitogenic and proliferative properties 35. However, in other cell types, for example, cartilage cells, the growth‐stimulating effects of IGF‐1 are GH‐independent 39. Furthermore, GH deficiency is the most common disorder seen in survivors of childhood cancer, and there are concerns regarding its use in treating cancer survivors as it might increase the risk of SPCs 40. Although IGF‐1 possesses antiapoptotic, cell survival, and transforming activities, it is not classed as an oncogene.

### Breast cancer

Findings to date on the role of IGF‐1 in breast cancer development vary depending on the study. An early case–control study conducted in 1993 demonstrated that circulating levels of IGF‐1 were higher in women with breast cancer compared to women without breast cancer 41. Since then, several epidemiological studies have reported that higher circulating levels of IGF‐1 are associated with an increased risk of breast cancer 22, 42, 43, 44, 45. This may arise as higher levels of IGF‐1 are associated with acceleration of early carcinogenesis 36. More recently, three meta‐analyses demonstrated positive associations between IGF‐1 and risk of breast cancer among premenopausal, but not postmenopausal women 46, 47, 48. A study by Schernhammer et al. aims to explain these observations by showing that premenopausal women with high IGF‐1 levels were at risk of higher IGF‐1R activation in mammary epithelial cells, which is suggested to increase survival of these cells with accumulating DNA damage, thereby facilitating stepwise carcinogenesis 49. These results may indicate the importance of IGF‐1 levels in younger women in early life or its possible interaction with other hormones such as estrodial 36 and growth hormone 39. In contrast to these studies, a large prospective study pooling two Swedish cohorts found no association between circulating IGF‐1 and risk of breast cancer, regardless of menopausal status 50. It is unclear why there is such a discrepancy in study findings. However, differences in findings may be due to timing of blood sampling, the patient cohort or the subset of breast cancer. Prospective studies are advantageous over retrospective studies since blood samples to measure IGF‐1 levels are collected before the clinical diagnosis of cancer and hence reverse causation (i.e., effects of an undiagnosed cancer on levels of IGF‐1) is less likely to play a role 50.

Estrogen plays an important role in the etiology of breast cancer, and there are experimental studies reporting cross‐talk between IGF‐1 and the estrogen receptor (ER) in mammary cells, possibly through synergistic effects that contribute to breast carcinogenesis 44, 51. Stewart et al. showed that estrogen increases IGF‐1 binding and IGF‐1R mRNA levels in the estrogen‐sensitive MCF‐7 cell line by 7‐ and 6.5‐fold, respectively 52. This suggests that one potential mechanism by which estrogen stimulates breast cancer cell proliferation may involve sensitization of IGF‐1 52.

Thus far, epidemiological evidence overall suggests a positive association between IGF‐1 and breast cancer risk, particularly in premenopausal women. Moreover, experimental evidence suggests that a link between IGF‐1 and estrogen may explain this positive association, but perhaps only in breast cancers that express the estrogen receptor.

### Lung cancer

Studies to date have investigated the association between IGF‐1 and lung cancer. Several studies have shown that circulating IGF‐1 levels were not associated with an increased risk of lung cancer 53, 54, 55, 56, 57. One case–control study found a positive association between IGF‐1 and risk of lung cancer (OR: 2.06; 95% CI: 1.19–3.56) 21. Furthermore, this study identified that the levels of IGF‐1 and IGF‐2 in plasma were not influenced by cigarette smoking 21.

IGFBP‐3 is the main IGF‐1‐binding protein in blood. IGFBP‐3 is generally considered to act as a tumor suppressor gene by reducing the ability of IGF‐1 to promote cell survival and proliferation 58. Although epidemiological studies overall found no association for IGF‐1, a reduced risk of lung cancer is reported with higher circulating levels of IGFBP‐3, when comparing the highest quartile versus lowest quartile of IGFBP‐3 in a Chinese prospective study (OR: 0.50, 95% CI: 0.25–1.02) 55. Moreover, several meta‐analyses have also reported an inverse association between IGFBP‐3 and risk of lung cancer 58, 59.

It is possible that both IGF‐1 and IGFBP‐3 contribute to the development of lung cancer. Cell culture studies have found that lung cancer cell lines, regardless of their histological subtypes, have the capacity to express IGF‐1 and its binding protein, IGFBP‐3, both in tumors and blood 60, 61.

Thus, until now, there is little evidence for a link between IGF‐1 and lung cancer risk, but an inverse association between IGFBP‐3 and lung cancer risk has been observed. These epidemiological observations are consistent with experimental data, which demonstrates that IGFBP‐3 block the mitogenic and antiapoptotic effects of IGF‐1 on lung cancer cells 21, 55.

### Prostate cancer

Associations between prostate cancer and IGF‐1 have been studied extensively, and consistently show a positive association. Since 1993, it has been investigated whether higher circulating IGF‐1 levels are associated with an increased risk of prostate cancer 62, 63. Early studies failed to demonstrate an association between IGF‐1 and prostate cancer risk. The first significant positive association between IGF‐1 and prostate cancer was examined in a case–control study by Mantzoros et al. By comparing men with prostate cancer to healthy controls, the odds ratio per 60 ng/mL increment in circulating levels of IGF‐1 was 1.91 (95% CI: 1.00–3.73) 64. Furthermore, the authors also mentioned that this association is further reinforced by the lack of association between IGF‐1 and benign prostatic hyperplasia.

**Figure 2:**
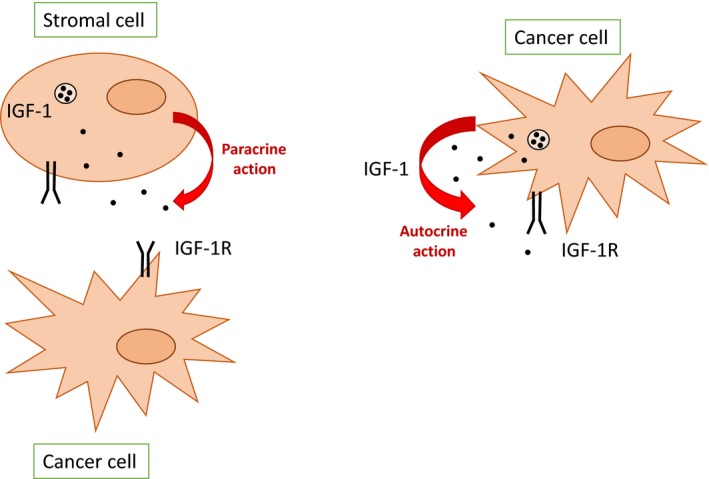
The autocrine and paracrine actions of IGF‐1.

IGF‐1 is known to stimulate the growth of prostate cancer cells by inducing cell proliferation and inhibiting apoptosis 65. The effect of IGF‐1 on prostate cancer cell lines has been extensively explored. For example, in vivo studies have shown significantly reduced proliferation rates in PC‐3 prostate cancer cell lines in IGF‐1‐deficient hosts, compared to control hosts 66. Exogenous IGF‐1 increased the invasive potential of the DU145 prostate cancer cell line, which was dependent on IGF‐1R, the ERK MAPK pathway, and the PI3K pathway 67. Furthermore, prostate cancer epithelial cells can stimulate their own growth by synthesizing and responding to IGF‐1 in an autocrine manner (Fig. 2), as opposed to paracrine signaling 68.

Overall, epidemiological and experimental evidence to date suggest a positive association between circulating IGF‐1 and prostate cancer risk.

### Colorectal cancer

Overall, studies have provided data showing that colorectal cancer is positively associated with IGF‐1 levels 23, 69, 70, 71, 72. Early studies in the late 1990s, suggested that high circulating IGF‐1 concentrations are associated with an increasing risk of colorectal cancer 23, 73. Nomura et al. evaluated the association between IGF‐1 and colon and rectal cancer separately 69. They showed a higher risk of colon cancer for those with IGF‐1 levels in the third (137–174 ng/mL) and fourth quartiles (IGF‐1 > 174 ng/mL) as compared to the controls (OR of 2.2 and 1.8, respectively). There was no association between IGF‐1 and rectal cancer. However, another study found a decreased risk of rectal cancer with high levels of circulating IGF‐1 70. The authors commented that this may be due to rectal cancer presenting at an earlier stage than colon cancer, which may have masked the association with IGF‐1 levels 70. On the other hand, data from two nested case–control study showed no statistically significant association between IGF‐1 and colorectal cancer risk 74, 75.

The epidemiological data on IGF‐1 and colorectal cancer is supported by laboratory evidence. The IGF‐1R is expressed in both normal colonic mucosa and human colorectal cancers 70. IGF‐1 has been speculated to cause proliferation of colorectal cancer cells and promote overexpression of the IGF‐1R in several studies, with no uniform consensus 76, 77, 78, 79. Furthermore, in acromegaly, a condition that is associated with high IGF‐1 levels, studies have shown that there is increased proliferation of normal colonic epithelium with an increased risk of developing colorectal adenomas and cancers in acromegaly patients 80, 81.

In colorectal cancer, the circulating levels of IGF‐1 is particularly influenced by nutritional status. Therefore, further research is required to investigate the role of obesity, ethnicity, and dietary habits possibly as confounders to IGF‐1 and colorectal cancer risk.

## IGF‐1 in Second Primary Cancers

This section summarizes studies on the emerging role of IGF‐1 in the development of SPCs with a focus on the definition of SPC as well as their potential link with IGF‐1 in the case of patients with primary breast, lung, prostate, and colorectal cancer.

SPCs are defined as malignant tumors diagnosed at the same time as the primary tumor or later, which are in a different organ 82 and are not a metastasis or recurrence of the original primary cancer 83.

### Breast cancer

The risk of developing a second primary breast cancer in a patient diagnosed with a first breast cancer has been studied extensively. It has been shown that in women with breast cancer, the risk of developing a new primary breast cancer in the contralateral breast is much higher than for healthy women developing a first breast cancer 84, 85. Only a small portion of this large risk is attributable to effects of treatment: lifestyle and genetic factors also need to be taken into account 84, and possibly the role of IGF‐1 in increasing breast cancer risk.

Breast cancer is also a common SPC in itself. For instance, it is the most common SPC that develops in young women treated for Hodgkin's lymphoma with supradiaphragmatic irradiation 86. The estimates of cumulative risks of developing breast cancer ranged from 35% by 40 years of age 87 to 48% by 40 years of age 88 after treatment. Much of this variation may be due to the differences in the age of diagnosis of Hodgkin's lymphoma during radiation treatment, or the duration and dose of treatment 88, 89. Even in the absence of treatment effects, in general, younger women are at a greater risk of developing a second primary breast cancer than older women 90, 91. For instance, a cohort study by Hancock et al. which reviewed women treated for Hodgkin's disease between 1961 and 1990 (mean follow‐up, 10 years) concluded that the greatest risk was seen in young women treated before the age of 15 years (RR: 136, 95% CI: 34–371), with a significant decline in relative risk with advancing age (above 30 years of age, RR: 0.7, 95% CI: 0.2–1.8) 92.

From a biological point of view, the pubertal growth of the mammary gland is mediated predominantly by the actions of IGF‐1 and GH via estrogen 93. It is postulated that this mechanism may make younger women more prone to developing a secondary breast cancer due to the increased levels of both IGF‐1 and estrogen during puberty, and the promoting effects of IGF‐1 41, 94. According to the evidence from in situ hybridization, in breast cancer, IGF‐1 is predominantly expressed in the stromal cells (mainly fibroblasts) and very rarely in the breast epithelium 28, 95. This supports the concept of a paracrine role of IGF‐1 in breast cancer (Fig. 2). It is possible that there is also an endocrine role, given that circulating IGF‐1 in the bloodstream is implicated in the malignant transformation of breast tissue 28, 43, 96. IGF‐1 functions to protect breast cancer cells from apoptosis and induces survival 28, suggesting that locally synthesized IGF‐1 influences the growth of human breast cancer cells. It can therefore be hypothesized that IGF‐1 produced by the stromal cells is increased in breast cancer (Fig. 2). This may then promote growth of a second primary breast cancer by entering the blood stream and acting in an endocrine fashion 94, 97.

### Lung cancer

The lungs are often regarded as one of the most common organs to develop a SPC 11, 98, 99. Common causes of a secondary lung cancer include a resected primary lung cancer, treatment‐related complications in breast cancer and head and neck cancers 99, 100, 101, as well as continued smoking 102, 103. In a US study, Johnson reported a 2–14% risk of developing a second lung cancer per person per year, with the risk increasing from twofold to sevenfold after 10 years of initial lung cancer diagnosis 104.

Head and neck cancer patients are at an increased risk of developing lung cancer with a standardized incidence ratio (SIR) of 3.75 (95% CI: 3.01–4.62) 98. In addition, according to data from the Surveillance, Epidemiology, and End Results (SEER) registries, approximately 5% of breast cancer survivors are diagnosed with a second primary lung cancer 11. Additionally, treatment of breast cancer patients with radiation postmastectomy has been shown to approximately double the risk of second primary lung cancer, especially in the ipsilateral lung 99. The rates of second primary lung cancer among women diagnosed with breast cancer before the age of 50 years is rising significantly, with the increase being as early as 1 year after breast cancer diagnosis 105. Radiotherapy treatment for breast cancer seems unlikely as the sole cause of this rise (see methodological section) because a long‐term latency period (5–10 years) is usually associated with radiotherapy treatment 105; proteins or hormones such as IGF‐1 should also be considered as predisposing factors.

When considering the biological effects of IGF‐1 in second primary lung cancer, it has been shown that lung mesenchymal cells locally synthesize IGF‐1 which acts on the bronchial epithelium in a paracrine manner 56. A possible mechanism explaining the role of IGF‐1 in second primary lung cancer is that higher IGF‐1 levels detected in lung cancer are probably regulated by the levels of tissue‐derived IGF‐1, but not circulating IGF‐1 106.

### Prostate cancer

The risk of all SPCs following a diagnosis of a first primary prostate cancer has been studied with variable results. In 1999, a Swiss study based on data collected from the Cancer Registries of the Swiss Cantons of Vaud and Neuchâtel investigated the risk of SPCs in prostate cancer survivors between 1974 and 1994. They found a significantly reduced incidence rate of all cancers in men diagnosed with prostate cancer, compared with the general population (SIR: 0.7, 95% CI: 0.6–0.8) 107. More recently, in 2014, a cohort study from the Swiss Canton of Zurich investigated the risk of SPCs in prostate cancer survivors between 1980 and 2010 19. They found an increased risk of SPCs among men with prostate cancer, compared to the general population (SIR: 1.11, 95% CI: 1.06–1.17). The inconsistency between these two studies may be due to the diagnosis of prostate cancer at an advanced stage with shorter survival in the earlier years of study 19. Therefore, the chance of developing a SPC was lower than what it is currently, when prostate cancer is generally diagnosed at a less advanced stage.

When looking at specific cancer types, Davies et al. reported that survivors of prostate cancer had a 40% lower risk of developing a SPC compared to the general male US population; the risk was lower for leukemias and cancers of the oral cavity, stomach, colon, liver, lung, and pancreas 108. However, they observed a higher risk of developing bladder 109, 110, renal, and endocrine cancers 111, 112; this seems to be influenced by pelvic radiation therapy for prostate cancer 108. Moreover, diagnostic bias is thought to play a role due to anatomy. However, Chrouser et al. did not observe an increased risk of bladder cancer after radiotherapy for prostate cancer 113, and there are some uncertainties in relation to the possible mechanism for the lack of association observed in this study. It is possible that there may have been an increased risk in this study that was not detected due to a short mean follow‐up period of 7.1 years or likelihood of underreporting SPCs. Based on the current evidence, it seems that the risk of developing a SPC after prostate cancer is higher, particularly for other urological cancers.

Prostate cancer is also commonly observed as a SPC in itself. Kok et al. concluded that in the first year following a first cancer diagnosis, male cancer survivors have a 30% increased risk of developing prostate cancer as a SPC, partly due to increased diagnostic activity of the urological organs or incidental finding following health check‐ups 114. Other studies have also shown an excess risk of developing prostate cancer as a SPC after a diagnosis of a bladder cancer as a first cancer 115, 116. In addition, cancer survivors diagnosed with a first primary urological cancer may request for prostate‐specific antigen (PSA) testing as a consequence of anxiety or persisting urological symptoms 114. Furthermore, survivors of melanoma are also at increased risk of developing prostate cancer 117.

In contrast to the biological mechanisms of breast and lung cancer, prostate cancer epithelial cells can stimulate their own growth by synthesizing and responding to IGF‐1 65, 68. Furthermore, there is evidence that IGF‐1 enhances the adhesion of prostate cancer cells and this promotes prostate cancer metastasis, possibly through the actions of IL‐17 118. The potential data does not suggest a direct causative role for IGF‐1 signaling in the progression and invasiveness of prostate cancer. The IGF‐1 pathway activates a number of downstream signaling pathways, including the phosphatidylinositol‐3 kinase (PI3‐K) pathway, the protein kinase C pathway, the CREB pathway, and the mitogen‐activated protein kinase (MAPK) pathway. These pathways contribute to prostate cancer through deregulation and constitutive activation of the pathway 67. While the etiology of IGF‐1 in second primary prostate cancer is unknown, it is plausible that those who develop prostate cancer may possess a common genetic, hormonal, or environmental factor that protects them from developing a SPC 119. Prostate cancer survivors have a lower risk of developing cancers of the stomach, lung, and pancreas 108, 120, raising the question of whether these patients are “protected” against these malignancies, or whether it is simply that they are above the age at which the risk of these tumors typical peak, which is at an earlier age 119.

### Colorectal cancer

Several studies have demonstrated an increased risk of developing secondary colorectal cancer following radiotherapy exposure, in particular, rectal cancer following radiation for prostate cancer and colorectal cancer following abdominopelvic radiation for cervical cancer. Brenner et al. investigated the risk in prostate cancer patients who underwent radiotherapy or surgery and reported a significantly increased risk of rectal cancer in the radiotherapy group, particularly for long‐term survivors, when comparing with the surgery group 121. Furthermore, Baxter et al. observed a significant increase in the development of rectal cancer postradiation for prostate cancer 122. However, radiation did not promote development of cancer in the remainder of the colon, suggesting that the effect of radiation is limited directly to irradiated tissue. In addition to prostate cancer patients, cervical cancer patients also seem to be at risk of developing colon cancer, as observed by Chatruvedi et al. 123.

In normal colonic tissue, IGF‐1 binds with high affinity to the IGF‐1R and activates specific insulin receptor substrates, which can modulate several downstream pathways involved in gene transcription, cell proliferation, and apoptosis 124. Although the etiology of IGF‐1 in second primary colorectal cancer is unknown, based on findings from normal colonic tissues, we can speculate about the potential complexity of this carcinogenic mechanism. With the exposure to radiotherapy, one hypothesis suggests that in individuals with higher IGF bioactivity, there is enhanced survival of partially transformed cells which leads to a larger pool of targets for subsequent “hits” initiating colorectal carcinogenesis via the process of stepwise carcinogenesis and malignant transformation. A second hypothesis suggests that the time needed for the progression of a fully transformed cell to fully developed cancer is inversely associated with IGF bioactivity 69.

### Methodological considerations for epidemiological studies investigating the link between IGF‐1 and risk of second primary cancers

In the clinical setting, it may be problematic to absolutely define whether the second tumor is in fact a SPC or a recurrence or a metastasis, and a definitive diagnosis may only be possible histologically. Whether the results showed in this review were strictly according to the standard are unclear, so therefore we need to consider the results with caution.

Even though several studies suggest a link between IGF‐1 and development of SPCs, several methodological issues need to be considered when assessing these epidemiological findings. Firstly, diagnostic bias may occur when the SPC is the main outcome of interest, as it may be detected following a diagnostic intervention related to the first primary tumor 115. Aside from diagnostic activity, treatment related to this first primary tumor may also increase the risk of developing a second primary tumor (e.g., chemotherapy and radiation therapy) 115, 125.

Secondly, when evaluating the effect of IGF‐1 on SPCs, one has to consider sources of errors that cause misclassification of this biomarker. Nondifferential misclassification of IGF‐1 may occur due to laboratory errors (e.g., specimen collection, processing, and storage) or changes in IGF‐1 assays 126. In addition, a single measurement of IGF‐1 may not reflect the actual underlying levels. Repeated measurements would reflect long‐term exposure and may be useful in the context of carcinogenesis 127. Aside from misclassification of data for IGF‐1, it is also possible to have misclassification related to the SPCs because it is not always possible from a pathological point of view to make a distinction between local recurrences, metastases, or a true SPC.

Thirdly, when studying the association between IGF‐1 and SPCs, one has to be aware of potential confounders such as smoking or treatment. In the case of lung cancer, the effect of current and past smoking needs to be removed to maximize the efficiency of the study 56 as some studies have shown that smoking decreases the levels of IGF‐1 128, while others have found no relationship 129. It is therefore possible that cigarette‐related carcinogen exposure may overshadow the more subtle effects of IGF‐1 on cancer development, which could explain the general lack of an association between IGF‐1 and risk of lung cancer 54. As a result, smoking may have an effect on IGF‐1 levels as well as the risk of developing a SPC, but it is unlikely to be an intermediate in the pathway between IGF‐1 and SPCs 130. Furthermore, treatment received for the primary cancer may confound the association between IGF‐1 and SPCs 114, 130. Similarly to smoking, treatment may also alter the effect of IGF‐1 as well as the risk of developing a SPC, but it is again unlikely to be an intermediate in the pathway between IGF‐1 and SPCs 130.

## Conclusion

In spite of a consistent positive observation between IGF‐1 and risk of first primary cancers (especially breast, prostate, and colorectal), the evidence for the role of IGF‐1 in the development of SPCs is less clear. Some of the evidence we gathered came from targeting the IGF system in cell culture studies, and therefore, we need to see the results with caution on whether it can be compared to clinical situations. However, the relevant influences of these pathways in SPCs are unknown. This lack of an association may be partly explained by methodological issues. With respect to the biological pathway, there is consistent evidence for the mitogenic role of IGF‐1 in carcinogenesis by increasing cell proliferation and inhibiting apoptosis. However, experimental studies highlight uncertainties regarding the role of IGF‐1 in the development of SPCs. More observational studies are needed to further understand the role of IGF‐1 in the development of specific SPCs, as well as to determine which pathways downstream of the IGF‐1R are involved in this process.

## Conflict of Interest

None declared.
